# Spatiotemporal Dynamics of High-Gamma Activities during a 3-Stimulus Visual Oddball Task

**DOI:** 10.1371/journal.pone.0059969

**Published:** 2013-03-21

**Authors:** Yoritaka Akimoto, Akitake Kanno, Toshimune Kambara, Takayuki Nozawa, Motoaki Sugiura, Eiichi Okumura, Ryuta Kawashima

**Affiliations:** 1 Department of Functional Brain Imaging, Institute of Development, Aging and Cancer, Tohoku University, Sendai, Japan; 2 Faculty of Environment and Information Studies, Keio University, Kanagawa, Japan; 3 Smart Ageing International Research Center, Institute of Development, Aging and Cancer, Tohoku University, Sendai, Japan; 4 International Research Institute of Disaster Science, Tohoku University, Sendai, Japan; 5 Department of Epileptology, Tohoku University Graduate School of Medicine, Sendai, Japan; 6 Division of Developmental Cognitive Neuroscience, Institute of Development, Aging and Cancer, Tohoku University, Sendai, Japan; University of British Columbia, Canada

## Abstract

Although many studies have investigated the neural basis of top-down and bottom-up attention, it still requires refinement in both temporal and spatial terms. We used magnetoencephalography to investigate the spatiotemporal dynamics of high-gamma (52–100 Hz) activities during top-down and bottom-up visual attentional processes, aiming to extend the findings from functional magnetic resonance imaging and event-related potential studies. Fourteen participants performed a 3-stimulus visual oddball task, in which both infrequent non-target and target stimuli were presented. We identified high-gamma event-related synchronization in the left middle frontal gyrus, the left intraparietal sulcus, the left thalamus, and the visual areas in different time windows for the target and non-target conditions. We also found elevated imaginary coherence between the left intraparietal sulcus and the right middle frontal gyrus in the high-gamma band from 300 to 400 ms in the target condition, and between the left thalamus and the left middle frontal gyrus in theta band from 150 to 450 ms. In addition, the strength of high-gamma imaginary coherence between the left middle frontal gyrus and left intraparietal sulcus, between the left middle frontal gyrus and the right middle frontal gyrus, and the high-gamma power in the left thalamus predicted inter-subject variation in target detection response time. This source-level electrophysiological evidence enriches our understanding of bi-directional attention processes: stimulus-driven bottom-up attention orientation to a salient, but irrelevant stimulus; and top-down allocation of attentional resources to stimulus evaluation.

## Introduction

Attention is important for various cognitive functions, such as selection of visual information and attentional resource allocation. The oddball paradigm, wherein stimuli are presented with different probabilities, has been frequently used to study the mechanism of attention. The 3-stimulus oddball paradigm includes two different infrequent stimuli, namely, the target and the infrequent non-target in addition to one frequent stimulus. It has been hypothesized that the processing of the target stimulus entails top-down allocation of attentional resources for stimulus evaluation [1], [2], while the processing of the infrequent non-target stimulus involves stimulus-driven bottom-up attentional orientation to a salient but irrelevant stimulus [3], [4]. Previous functional magnetic resonance imaging (fMRI) studies revealed the involvement of the occipital regions in the processing of infrequent non-target stimuli [4], [5], and the involvement of the middle frontal gyrus (MFG), intraparietal sulcus (IPS), and thalamus during target detection [5]–[7]. These regions are involved in the cortical circuits that guide bottom-up and top-down attention [8]. A previous fMRI study also revealed that functional connectivity within attention network is linked to behavioral performance, i.e., reaction time [9].

Although fMRI has excellent spatial resolution, it has limited time resolution, due to the slow hemodynamic response. On the other hand, electrophysiological measurements, such as electroencephalogram (EEG) or magnetoencephalography (MEG) provide very good temporal resolution. It is well known that infrequent stimuli in the oddball paradigm elicit the P300 event-related potential (ERP), which contains at least two distinguishable subcomponents, namely, P3a and P3b. P3b is elicited by infrequent target stimuli, typically 300 to 600 ms after the stimulus onset [10]. P3a is elicited by infrequent non-target stimuli, and often occurs more quickly than P3b [11]. In addition to ERP, event-related synchronization (ERS), especially in the gamma band, is also important for attention [12]. Gamma ERS shows a close spatial correspondence with fMRI activations [13], and is believed to reflect neuronal processing or local encoding [14], [15]. Attention modulates gamma ERS in the early visual areas [16] and the gamma ERS in the visual cortex predicts the speed of change detection [17]. Long-range gamma band phase synchronization has been also reported for various cognitive tasks [18], [19] and is believed to reflect the neuronal communication that subserves various cognitive functions [20].

Gamma ERS also provides temporal information on time resolution comparable to that of ERP. In addition, focusing on the rhythmic activity enables assessing functional connectivity between distant brain regions. Therefore, it is expected that investigating the gamma activity will link the findings from fMRI and ERP studies and extend our understanding about the neural basis of top-down and bottom-up attention. There are several studies reporting gamma ERS elicited during oddball tasks [21]–[27]. Lee et al. determined that the generator of EEG high-gamma (65–85 Hz) ERS is localized in the MFG [27]. In addition, gamma band coherence among anterior and posterior electrodes was reported in EEG channel-level studies [28], [29]. However, understanding of the temporospatial brain activity is still incomplete, and moreover, source-level electrophysiological functional connectivity during the oddball task has not yet been reported. This is worth studying because it enables us to test whether the strength of electrophysiological functional connectivity within the attention network increases during the oddball task, and if so, whether this increase predicts behavioral performance.

The purpose of this study was to investigate the temporospatial dynamics of electrophysiological brain activity associated with top-down and bottom-up attentional processes. We focused on the high-gamma activities (52–100 Hz), because it appears to have more specific timing and localization than those of lower frequency [30], [31]. This study complements earlier fMRI studies by providing information about the temporal properties of brain activities. We used MEG with dual-state adaptive spatial filtering [32] and imaginary coherence analysis in source-space [33]. The dual-state adaptive spatial filtering technique results in increased fidelity of higher frequency source reconstruction, as compared to the traditional adaptive spatial filter technique. In the traditional filter technique, weights are computed from unfiltered or wideband data and are therefore inherently biased toward resolving low-frequency brain activity [32]. Imaginary coherence analysis is one of the functional connectivity analyses and can remove spurious coherence caused by non-interacting sources [34], [35]. We also examined the coherence of theta band activity because theta band oscillation plays a role in attentional processing [36] and modulates gamma activity in a phasic manner [37]. Theta activity is also reported to increase together with P300 during the oddball paradigm [38].

## Materials and Methods

### Participants

Fourteen healthy, right-handed participants (9 males, 5 females) took part in this study. The mean age of the subjects was 21 years (age range 18–25 years). In accordance with the Declaration of Helsinki (1991), written informed consent was obtained from each subject. This study was approved by the Ethics Committee of Tohoku University Graduate School of Medicine. Handedness was evaluated using the Edinburgh Handedness Inventory [39]. All subjects had normal or corrected to normal vision, and none had a history of neurological or psychiatric disease.

### Three-stimulus Oddball Task

**Figure 1:**
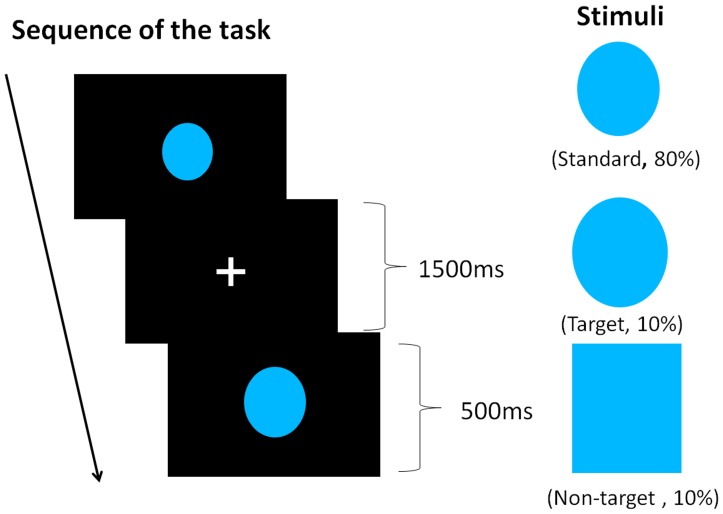
Experimental paradigm of 3-stimulus oddball task.

We used a 3-stimullus oddball task, using the same stimulus as Walhovd et al. [40] (Fig. 1). In this task, target, non-target, and standard stimuli were presented with an appearance rate of 10%, 10%, and 80%, respectively. Each stimulus was presented for 500 ms. Inter-stimulus intervals were 1500 ms. There were 260 trials in all. Subjects pushed a button with their right index finger only when the target stimulus appeared. The standard stimuli were blue elliptic shapes, the height of which subtended a visual angle of 4.6° and the width of which subtended a visual angel of 4.1°. Target stimuli were larger blue elliptic shapes, the height of which subtended a visual angle of 5.6° and the width of which subtended a visual angle of 4.6°. The infrequent non-target stimuli were large blue rectangles, the height of which subtended a visual angle of 6.4° and the width of which subtended a visual angle of 5.6°. The field of vision angle was considered to be central eye field to prevent eye movement. Viewing distance was 35 cm.

### DATA Acquisition

MEG data were acquired using a whole-head 200-channel MEG system (PQA160C, Yokogawa Electric Corporation, Japan) with a sample rate of 1000 Hz and with a low-pass 100 Hz filter. The head shape of each participant was digitized using a 3D digitizer (FastSCAN Cobra, Polhemus Inc., USA) and co-registered with individual structural MR images. Structural MR images were acquired using a 3T MR system (Achieva, Philips, Netherlands).

### Preprocessing

The Fieldtrip software package (http://fieldtrip.fcdonders.nl) was used for independent component analysis (ICA) of MEG data and typical noise components (e.g., eye-blink) were removed by a researcher based on visual inspection. The average number of the remaining ICs were 195.9 (SE = 0.7). The data were reconstituted from the ICs remaining after the removal of the artifactual ICs. We applied the array-gain-constraint version of diagonal loading (Tikhonov regularization; see Supplementary of Ueno et al. (2012) [41]) to all source reconstructions in this study. After this preprocessing, the data were band-pass filtered in the 52–100 Hz frequency domain. Trials were epoched from 300 ms before stimulus onset to 600 ms after stimulus onset. Trials with incorrect responses or magnetic flux in excess of 2000 fT in any channel were excluded. The number of analyzed trials was nearly equalized among conditions in order to produce similar noise levels across conditions [42]. This was done by randomly selecting the same number of standard trials and non-target trials for analysis.

### Localizing 3-stimulus Oddball Task-relevant High-gamma Activity

MEG data were analyzed using dual-state adaptive spatial filtering that was optimized for time-frequency source reconstructions from MEG or EEG data [32]. The entire brain was covered by virtual sensors with 5 mm voxels in MNI space. Functional images were created by calculation of the power increase relative to the baseline using a logarithmic conversion. The baseline period was defined as the time between −100 to 0 ms of the stimulus onset, and the active period was defined as the period between stimulus onset (0 ms) and 600 ms after stimulus onset. The active period was divided into 100 ms time windows. Images were normalized with a standard T1 template image and spatially smoothed with a 10 mm FWHM Gaussian kernel using SPM8 (Wellcome Department of Cognitive Neurology, London, UK).

Group analysis was conducted using SPM8. In order to avoid any biases in the selection of ROIs for analysis, we evaluated the task relevant regions of the 3-stimuli oddball task using F-contrast that included two factors. These factors were stimulus (target, non-target, and standard) and time window (0–100 ms, 100–200 ms, 200–300 ms, 300–400 ms, 400–500 ms, and 500–600 ms). This F-contrast tests whether any stimulus in any time-windows is different from zero. The statistical threshold at the voxel level was set at P<0.05 (FWE, correcting for spatial dimension at the voxel level). The peak voxels obtained from clusters containing more than 10 voxels were defined as regions of interest (ROIs).

### Time Course of High-gamma Power Change in ROIs

Firstly, we extracted the high-gamma power change in ROIs, using the time between −300 and 0 ms of the stimulus onset as the baseline to obtain stable results. We then compared the power change in each time window of interest between 0 and 600 ms with moving 100 ms windows for target stimuli to that for standard stimuli, and the power change for non-target stimuli to that for standard stimuli using paired t-tests. The statistical threshold was set at p<0.05, corrected for multiple comparisons using the FDR method [43] controlling the number of time windows, ROIs, and comparison pairs (i.e. target vs. standard, and non-target vs. standard).

### Imaginary Coherence Analysis

A serious problem in coherence analysis of EEG or MEG arises from nil coherence caused by non-interacting sources (i.e., coherence which reflects artifacts rather than brain interaction). To identify true brain interaction, Nolte et al. [34] proposed the use of the imaginary part of coherency, which is only sensitive to synchronizations of two processes that are time-lagged to each other. Because volume conduction and inversion leakages do not cause a time-lag, imaginary coherence is insensitive to artifactual coherence.

In the imaginary coherence analysis, we selected the ROIs located in the regions related to control of attention (i.e. frontal region, parietal region, and thalamus) as seed and searched for regions in which the target or non-target condition showed stronger imaginary coherence than the standard condition, assuming the whole brain as the search volume. For the analysis of the high-gamma band, we used the same time windows as the time-course analysis. For the analysis of the theta band, reconstituted data were band-pass filtered in the 4 Hz to 8 Hz frequency domain. The baseline was defined as −300 to 0 ms, and imaginary coherences in three time windows were statistically evaluated (0 to 300 ms, 150 to 450 ms, and 300 to 600 ms). The statistical threshold was set at p<0.05 (FWE), corrected for the spatial dimension at the voxel level using non-parametric mapping software (SnPM; http://go.warwick.ac.uk/tenichols/snpm). We used a non-parametric statistical test due to unknown nature of the data distribution. Only results that included more than 10 significant voxels are reported.

## Results

### Behavioral Results

The rate of correct responses to non-target, target, and standard stimuli were 99.7% (SE = 0.3), 84.9% (SE = 3.4), and 99.3% (SE = 0.3), respectively. The average reaction time to target stimuli was 543 ms (SE = 34.7). The average number of analyzed non-target-, target-, and standard-stimulus trials were 22.7 (SE = 0.89), 18.8 (SE = 0.87), and 22.7 (SE = 0.89), respectively.

### Localizing High-gamma Activity Relevant to the 3-stimulus Oddball Task

**Figure 2:**
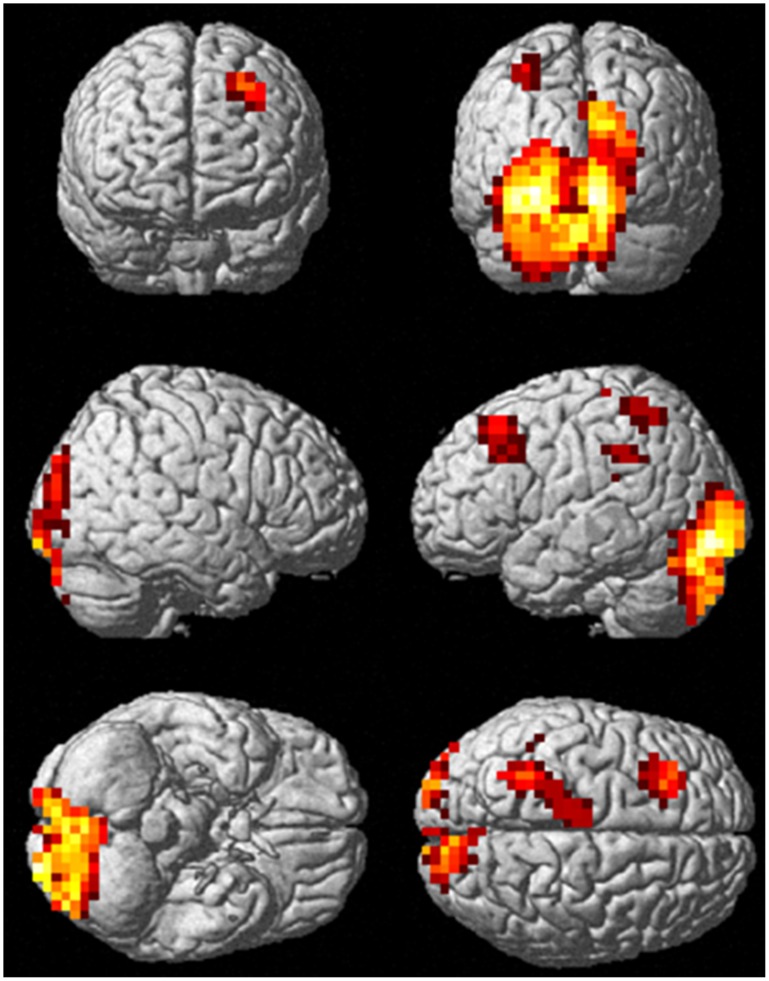
High-gamma activity relevant to a 3-stimulus oddball task (p<0.05, FWE corrected).

The F-contrast obtained from the 3-stimulus oddball task revealed task-relevant regions, including the bilateral occipital region, the left frontal region, the left parietal region, and the left thalamus (Fig. 2). The peak voxels were defined as ROIs for further analysis (Table 1).

**Table 1:** High-gamma activity relevant to a 3-stimulus oddball task (p<0.05, FWE corrected).

		MNI coordinate				
Region		x	y	z	*F*-value	P_FWE_corr_
Lingual gyrus	R	5	−96	−17	6.84	p<0.001
Middle occipital gyrus	L	−25	−86	−2	6.23	p<0.001
Inferior occipital gyrus	L	−20	−96	−7	6.19	p<0.001
Cuneus	R	10	−81	28	4.91	p<0.001
Thalamus	L	−5	−26	8	3.51	p<0.05
Middle frontal gyrus	L	−25	24	48	3.91	p<0.005
Intraparietal sulcus	L	−25	−46	53	3.84	p<0.005
Paracentral lobule	L	−10	−21	63	3.63	p<0.01
Supramaginal gyrus	L	−45	−41	33	3.49	p<0.05
Postcentral gyrus	L	−35	−31	38	3.44	p<0.05
Midcingulate cortex	L	−15	−31	48	3.28	p<0.05

### Time Course of High-gamma Power Change in ROIs

**Figure 3:**
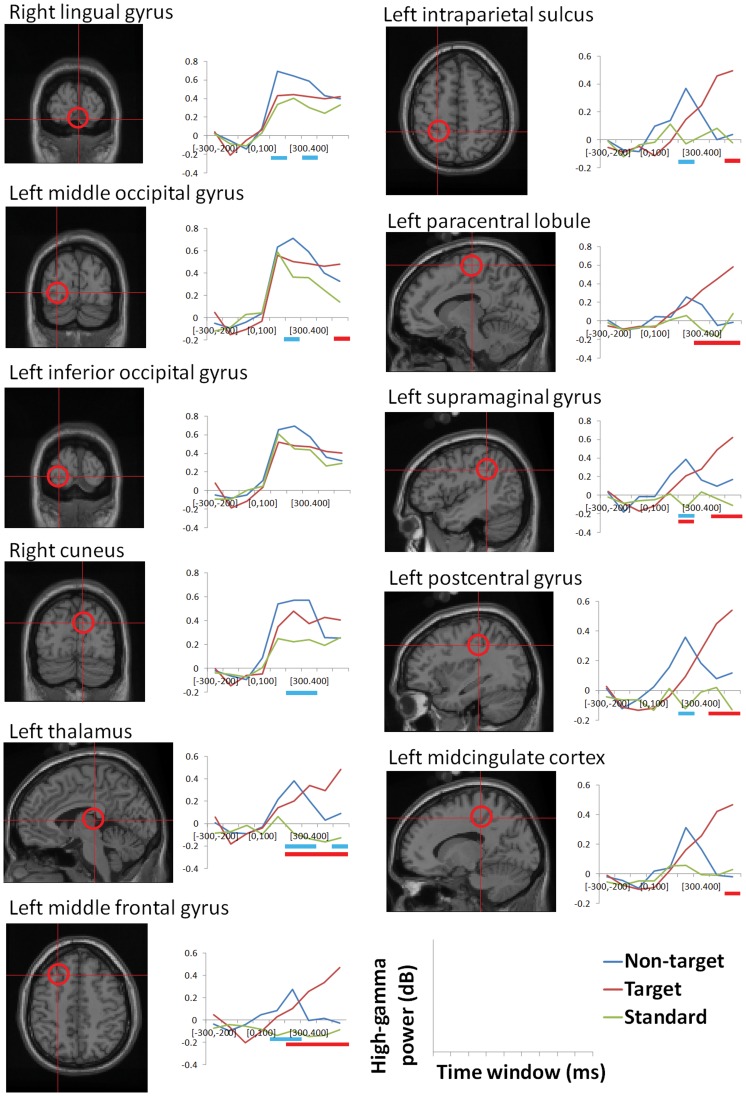
Time course of high-gamma power changes in each region of interest. Blue bars indicate significant differences between the non-target condition and the standard condition, while red bars indicate significant differences between the target condition and the standard condition (p<0.05, FDR corrected).

**Figure 4:**
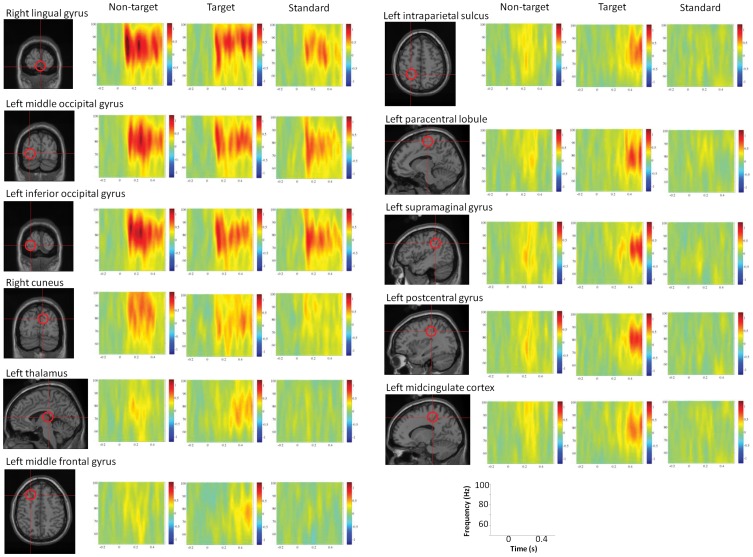
Time-frequency representation of high-gamma power changes in each region of interest. Warm colors indicate synchronization, while cold colors indicate desynchronization.

Results of time-course analysis of ROIs are shown in Figure 3. Time-frequency representations are shown in Figure 4. The earliest significant differences between the non-target and standard conditions were observed in the left MFG and in the right lingual gyrus at the time window of 100 to 200 ms. In the 200 to 300 ms time window, the left IPS, the left middle occipital gyrus, the right cuneus, the left thalamus, the left supramaginal gyrus, and the left postcentral gyrus were significantly different for non-target and target stimuli. On the other hand, the earliest significant differences between the target and standard conditions were observed in the left MFG, in the left thalamus, and in the left supramaginal gyrus at the time window of 200 to 300 ms. Significant differences were found between the target and standard conditions in the left paracental lobule after 300 ms and in the left postcentarl gyrus after 400 ms. At the time window of 500 to 600 ms, the left IPS, the left middle occipital gyrus, and the left midcingulate cortex were significantly different for the target and the standard conditions.

### Additional Response-locked Analysis

In order to examine the effect of motor response, we conducted additional response-locked analysis in the left IPS, the left supramaginal gyrus, and the left postcentral gyrus, which were located near the somatosensory area and showed significant effects not only in non-target-standard comparisons but also in the target-standard comparisons around the response time. We extracted the high-gamma power change from −800 to 100 ms of the response onset in the target condition. The baseline period was defined as the time between −800 to −500 ms.

**Figure 5:**
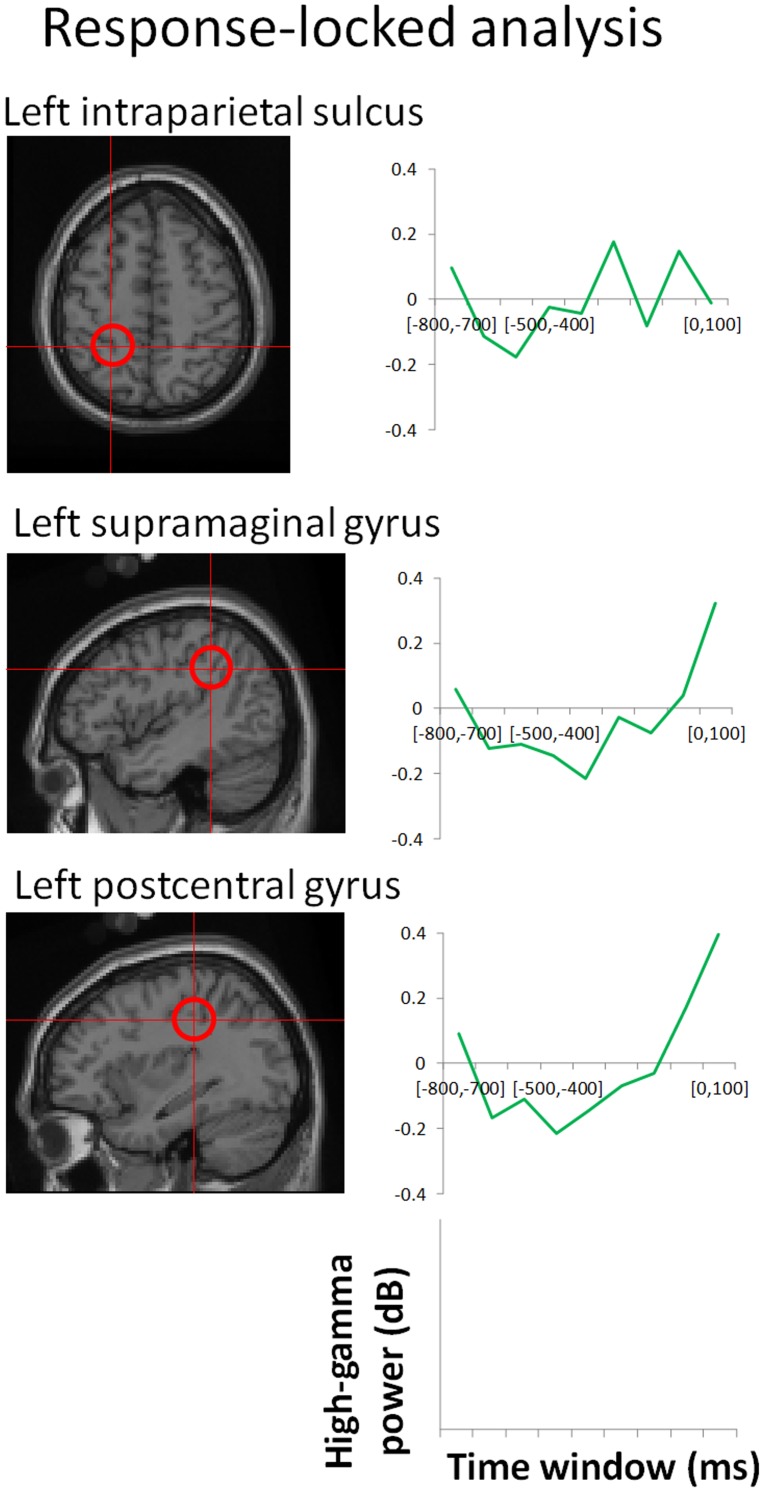
Time course of high-gamma power changes in response-locked analysis.

As a result, the left supramaginal gyrus and the left postcentral gyrus showed rapid increase of high-gamma ERS around the response onset (Figure 5). Although high-gamma ERS from 0 to 100 ms of response onset was not statistically significant in one sample t-test (*p = *0.17, *p = *0.10, respectively), we cannot perfectly exclude the possibility of the motor response effect in these ROIs. In contrast, the left IPS did not show an increase in high-gamma ERS around the response onset (*p = *0.50 at −100 to 0 ms, and *p* = 0.95 at 0 to 100 ms), suggesting that observed high-gamma ERS in the left IPS in the stimulus-locked analysis cannot be explained by the motor response alone.

### Imaginary Coherence Analysis

The results of imaginary coherence analysis are summarized in Table 2. The ROIs located in the left MFG, the left IPS, and the left thalamus were selected as seed. There was greater high-gamma imaginary coherence between the left IPS and the right MFG, and between the left IPS and the left postcentral gyrus in the 300 to 400 ms window of the target condition than in the standard condition. In the theta band, the left thalamus showed greater imaginary coherence with the left MFG in the non-target condition than in the standard condition from 150 to 450 ms.

**Table 2:** Results of imaginary coherence analysis for a 3-stimulus oddball task.

						MNI coordinate					
Seed (MNI coordinate)	frequency	time window	contrast	Region		x	y	z	*Pseudo*-tvalue	P_FWE_corr_	Clustersize(voxel)
Left intraparietal sulcus(−25, −46,53)	52–100 Hz	300–400 ms	Target vs.Standard	Middle frontalgyrus	R	45	49	18	5.74	p = 0.005	92
Left intraparietal sulcus(−25, −46,53)	52–100 Hz	300–400 ms	Target vs.Standard	Postcentralgyrus	L	−55	−11	58	5.30	p = 0.015	21
Left thalamus(−5, −26,8)	4–8 Hz	150–450 ms	Non-target vs.Standard	Middle frontalgyrus	L	−33	58	15	5.79	p = 0.008	13

### Correlation between Reaction Time and Coherence in the Attention Network

We examined whether the coherences in the attention network would predict individual differences in mean reaction time to target stimuli. Therefore, we examined the correlation between the reaction time and the strength of imaginary coherence among the left MFG, the right MFG, the left IPS, and the left thalamus. We calculated the correlations between the reaction time and the strength of imaginary coherence in each pair in each time window of interest between 200 and 600 ms with moving 100 ms windows, because the earliest significant difference between target and standard was found at 200 ms after the stimulus onset. We also examined the correlations between mean reaction time and regional high-gamma power.

**Figure 6:**
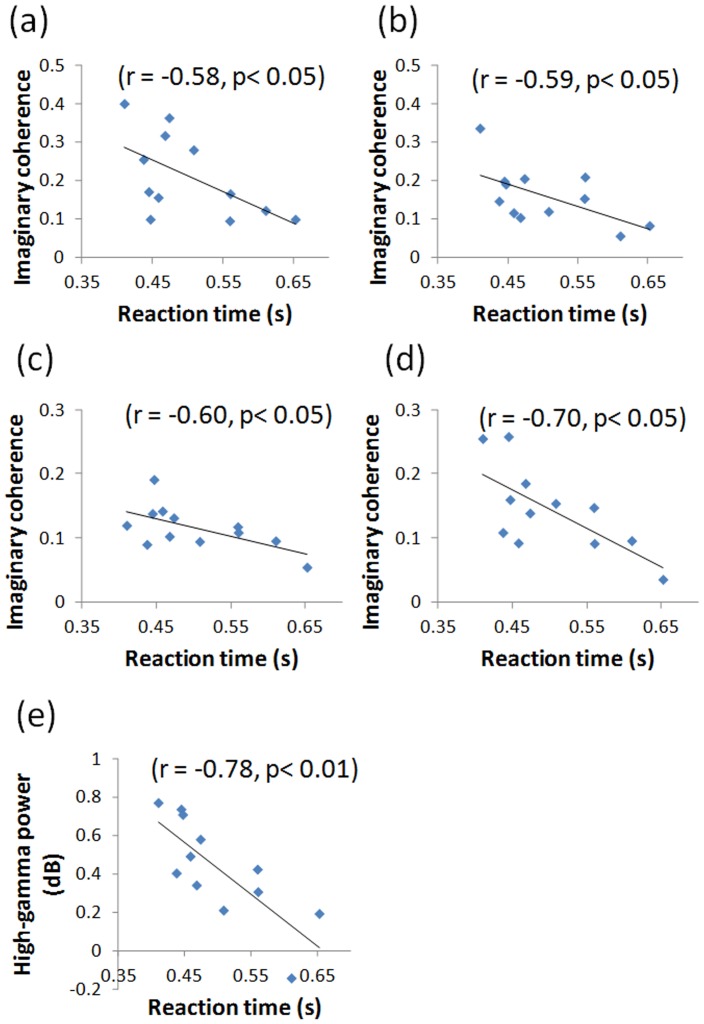
Significant correlation between reaction time and the strength of high-gamma imaginary coherence or regional high-gamma power. (a) Reaction time *vs.* the strength of high-gamma imaginary coherence between the left IPS and the left MFG during the time window of 300 to 400 ms and (b) during the time window of 400 to 500 ms. (c) Reaction time *vs.* the strength of high-gamma imaginary coherence between the left IPS and the left MFG during the time window of 400 to 500 ms and (d) during the time window of 500 to 600 ms. (e) Reaction time *vs.* the high-gamma power in the left thalamus during the time window of 300 to 400 ms.

Two subjects were excluded from the correlation analysis because their reaction times were extremely large (2 *SD*s above the mean). The results are summarized in Table 3. We found that reaction time was negatively correlated with imaginary coherence between the left MFG and the left IPS at the time window of 300 to 400 ms and at 400 to 500 ms, (*r* = −0.58, *p*<0.05; *r* = −0.58, *p*<0.05, respectively). Reaction time was also negatively correlated with imaginary coherence between the left MFG and the right MFG at the time window of 400 to 500 ms and 500 to 600 ms, (*r* = −0.60, *p*<0.05; *r* = −0.70, *p*<0.05, respectively). Reaction time was negatively correlated with the regional high-gamma power in the left thalamus at the time window of 300 to 400 ms (*r* = −0.78, *p*<0.01). The scatter plots are shown in Figure 6.

**Table 3:** Correlation coefficient between reaction time and the strength of high-gamma imaginary coherence or the high-gamma power.

	Time window (ms)			
	[200,300]	[300,400]	[400,500]	[500,600]
***Imaginary coherence***				
Left middle frontal gyrus - right middle frontal gyrus	−.34	−.27	−.60*	−.70*
Left middle frontal gyrus - left thalamus	−.43	−.43	−.46	.10
Left middle frontal gyrus - left intraparietal sulcus	.03	−.58*	−.59*	−.40
Right middle frontal gyrus - left thalamus	−.01	.34	−.46	−.46
Right middle frontal gyrus - left intraparietal sulcus	.13	−.09	.03	.11
Left thalamus - left intraparietal sulcus	−.24	−.52	−.26	−.04
***High-gamma power***				
Left middle frontal gyrus	−.31	−.33	−.29	−.05
Right middle frontal gyrus	.29	−.24	.01	.24
Left thalamus	−.36	−.78**	−.34	−.14
left intraparietal sulcus	.04	−.37	−.38	.20

* p<.05,

** p<.01.

## Discussion

We examined high-gamma activities during visual attentional processes using MEG with dual-state adaptive spatial filtering. Our results showed the involvement of the frontoparietal attention network and visual areas and clarified the temporal aspects of their activation. In addition, this is the first study to demonstrate source-level electrophysiological functional connectivity that underlies bottom-up and top-down attentional processes during a 3-stimulus visual oddball task. The strength of high-gamma coherences in attention network were linked to behavior performance (i.e. reaction time). The high-gamma power in the left thalamus was also correlated with the reaction time.

We found that several regions including the left MFG, the left IPS, and the left thalamus showed high-gamma ERS, both in the target and non-target conditions, which is consistent with previous fMRI studies. According to Corbetta et al. (2008) [44], the dorsal attention network is comprised of the bilateral IPS and the frontal eye field, but the left MFG is not involved. However, these authors argue that the prefrontal cortex is also a possible source of top-down signal. Indeed, several authors have reported that the left MFG activates during voluntary attentional control [6], [45]–[47]. Moreover, fMRI functional connectivity analysis suggests that the left MFG is a source of the top-down modulation of the visual association cortex [48]. We also observed increased high-gamma ERS in the right lingual gyrus and the right cuneus in the non-target condition, and increased high-gamma ERS in the left paracentral lobule and the left midcingulate cortex in the target condition. This is also reasonable because the lingual gyrus and the cuneus are a visual area, and they are therefore naturally involved in bottom-up attention. The paracentral lobule and the midcingulate cortex are motor-related areas. Therefore, the involvement of these areas in the target condition is not surprising because motor responses were included only in the target condition.

In terms of the temporal aspect of high-gamma ERS, our results indicated that the earliest significant effects were observed in the left MFG, the left thalamus, and the left supramaginal gyrus at the 200 to 300 ms time window in the target condition, and in the right lingual gyrus and the left MFG at the 100 to 200 ms time window in the non-target condition. These results are basically compatible with previous findings. For example, the earliest effect in MFG in the target condition was consistent with a study in which monkey prefrontal neurons reflected the target location faster than parietal neurons during top-down attention [49], and with fMRI studies that used effective connectivity analysis to demonstrate causal streams from frontal to parietal during attention holding tasks [47], [50]. The earliest effect in the thalamus in the target condition was also consistent with a deep brain stimulation study that showed that the thalamus supports the early recognition of targets [51] and with fMRI studies that showed the involvement of thalamus in higher-order mental processes, including attention [52], [53]. In the non-target condition, the earliest effect in the right lingual gyrus was also consistent with the nature of bottom-up attention. At first glance, frontal activities preceding parietal regions in the non-target condition appears somewhat inconsistent with the nature of stimulus-driven attention. Because our analysis focused on examining the entire brain activity, it might be not suitable to strictly determine the sequence of activities. For example, we analyzed brain activities by consecutive 100 ms time windows that started at 0 ms. This time window might not be useful for comparison of the earliest high-gamma activity in the frontal and parietal regions. This possibility is supported by the p-values of the statistical test between the non-target and the standard conditions during the 0 to 100 ms time window in the left MFG and the left IPS, both of which were marginally significant (*p* = 0.07 and *p* = 0.10, respectively).

Even if exact conclusions about difference in the processing sequence are not possible, we observed a clear difference in the temporal pattern of high-gamma ERS between top-down and bottom-up attention. In bottom-up attention, high-gamma ERS occurred quickly, even earlier than the time window of P3a, probably due to the perceptual salience of the non-target stimuli. However, high-gamma ERS decreased quickly because non-target stimuli are irrelevant to the current goal. In top-down attention, while high-gamma ERS started earlier than the time window for P3b, it was relatively slow. However, in contrast to the bottom-up attention, high-gamma ERS was sustained, or even increased, after reaching significance, probably due to the relevance of the stimulus to the current goal.

We also observed increased imaginary coherence in the high-gamma band between the left IPS and the right MFG in the target condition and in the theta band between the left thalamus and the left MFG in the non-target condition. Both of these observations are in accordance with the time windows associated with P300. Theta activity in frontal regions is associated with working memory [54], prediction errors [55], and also visual attention [36], [56]. Causal flow between the left thalamus and the left MFG during attention-holding tasks has also been reported in previous fMRI studies [47], [50]. It has also been proposed that the thalamus plays a role in regulating activities across cortical regions [31], [57], [58]. Thus, the observed theta band coherence may reflect these processes, although there is some controversy as to whether theta ERS and P300 reflect physiologically distinct mechanisms [59]. The right MFG is associated with the ventral network [44] and may link the dorsal and ventral networks [60]. The ventral network is strongly activated by stimuli that are important, even if they are not very distinctive [44]. The target stimuli in our study had these properties. Therefore, both the dorsal and ventral networks might be activated by the target. Based on this assumption, the observed imaginary coherence between the left IPS and the right MFG might reflect the linking process of the dorsal network with the ventral network.

Correlation analysis revealed that individual differences in functional connectivity within attention networks predicted individual differences in the speed of responses to target stimuli. All of the significant correlations were negative, indicating that individuals with higher coherence in attention networks exhibited faster responses. Although imaginary coherence analysis *per se* did not allow us to determine the direction of the information flow, we speculated that the left MFG was the upper stream, given the following temporal sequence: starting at the time window of 200 to 300 ms, there was increased high-gamma ERS in the left MFG; at 300 to 400 ms, the strength of imaginary coherence between the left MFG and the left IPS was negatively correlated with response time and the strength of imaginary coherence between the left IPS and the right MFG increased; at 400 to 500 ms, the strength of imaginary coherence between the left MFG and the right MFG negatively correlated with response time; and finally, at 500 to 600 ms, the left IPS increased high-gamma ERS. Based on this assumption, our results indicated that functional connectivity between the sources of top-down signal and the dorsal attention network (i.e. the left MFG - the left IPS) or the ventral attention network (i.e. the left MFG - the right MFG) predict behavioral performance, but those between the dorsal attention network and the ventral attention network do not (i.e. the left IPS - the right MFG). We observed significant correlations with the reaction time in coherence pairs in which only one of regions (i.e. the left MFG) showed significantly increased high-gamma ERS. This was interpretable because non-significance implies that there are considerable individual differences, and therefore they were observable. We also found a negative correlation between the reaction time and high-gamma power in the thalamus at the time window of 300 to 400 ms. As discussed previously, the thalamus plays an important role in attention modulation and behavioral response modulation. These results are in line with previous studies that indicate the amplitude of hemodynamic response in the MFG and that in the thalamus predict the response time of visual target detection [5].

We selected a similar high-gamma band (52 to 100 Hz) to that of previous studies [61], [62]. The ranges of reported frequencies of high-gamma varies widely among studies and include 59 to 85 Hz, 65 to 90 Hz, 70 to 85 Hz, 70 to 90 Hz, 75 to 100 Hz, and 81 to 101 Hz [63]. Recently, Belluscio et al. (2012) [64] reported different connectivity properties for 30 to 50 Hz, 50 to 90 Hz, and 90 to 150 Hz. Ray and Maunsell (2011) [65] also reported that frequencies above 80 Hz are mostly due to spiking whereas gamma activity of 30 to 80 Hz is rhythmic. Thus, it is possible that the band we chose might overlap these two bands and may not be well characterized. However, in most of the time-frequency representations shown in Fig. 4, there were oscillatory peaks around 80 Hz. Therefore, we think our results primarily reflect gamma rhythms. Indeed, the boundary between gamma rhythm and spikes should not be strict [65], given that the oscillation frequency of a network critically depends on excitation-inhibition balance [66].

A limitation of the present study is the small number of trials per condition per subject. We selected the number of infrequent stimuli based on the previous studies that indicated approximately 20 target trials stabilize the amplitude of P300 [67] and the response to novel stimuli are rapidly habituated with repetition [68]. However, the number of infrequent stimuli may have been low for analysis of ERS of the high-gamma band. Thus, our results of individual subject analysis should be considered as preliminary and even though the significant correlation obtained between the reaction time and the imaginary coherences seemed to be interpretable, future investigation is required.

### Conclusions

We investigated the spatiotemporal dynamics of electrophysiological activity related to attentional processes using MEG with a dual-state adaptive spatial filtering technique, which may increase fidelity in source reconstruction. We identified high-gamma event-related synchronization in the left middle frontal gyrus, the left intraparietal sulcus, the left thalamus, and the visual areas in different time windows for the target and non-target conditions. We also observed elevated high-gamma coherence between the left IPL and the right MFG in the target condition at 300 to 400 ms, which might reflect the linking process of the dorsal network with the ventral network. In addition, the strength of high-gamma imaginary coherence between the left MFG and left IPL, between the left MFG and the right MFG, and the high-gamma power in the left thalamus predicted the speed of target detection. This source-level electrophysiological evidence enriches our understanding of the bi-directional attention processes: stimulus-driven bottom-up attentional orienting to a salient but irrelevant stimulus, and top-down allocation of attentional resources to stimulus evaluation.
